# Re-identification of home addresses from spatial locations anonymized by Gaussian skew

**DOI:** 10.1186/1476-072X-7-45

**Published:** 2008-08-12

**Authors:** Christopher A Cassa, Shannon C Wieland, Kenneth D Mandl

**Affiliations:** 1 Children's Hospital Informatics Program, Children's Hospital Boston, Boston, MA, USA; 2 Computer Science and Artificial Intelligence Laboratory, Massachusetts Institute of Technology, Cambridge, MA, USA; 3 Harvard-MIT Division of Health Sciences and Technology, Cambridge, MA, USA; 4 Harvard Medical School, Boston, MA, USA

## Abstract

**Background:**

Knowledge of the geographical locations of individuals is fundamental to the practice of spatial epidemiology. One approach to preserving the privacy of individual-level addresses in a data set is to de-identify the data using a non-deterministic blurring algorithm that shifts the geocoded values. We investigate a vulnerability in this approach which enables an adversary to re-identify individuals using multiple anonymized versions of the original data set. If several such versions are available, each can be used to incrementally refine estimates of the original geocoded location.

**Results:**

We produce multiple anonymized data sets using a single set of addresses and then progressively average the anonymized results related to each address, characterizing the steep decline in distance from the re-identified point to the original location, (and the reduction in privacy). With ten anonymized copies of an original data set, we find a substantial decrease in average distance from 0.7 km to 0.2 km between the estimated, re-identified address and the original address. With fifty anonymized copies of an original data set, we find a decrease in average distance from 0.7 km to 0.1 km.

**Conclusion:**

We demonstrate that multiple versions of the same data, each anonymized by non-deterministic Gaussian skew, can be used to ascertain original geographic locations. We explore solutions to this problem that include infrastructure to support the safe disclosure of anonymized medical data to prevent inference or re-identification of original address data, and the use of a Markov-process based algorithm to mitigate this risk.

## Background

To develop broadly integrated national healthcare information infrastructure, the utility of sharing personally identifiable data for clinical care, public health and research must always be weighed against privacy concerns. For example, automated outbreak detection systems for surveillance of influenza and bioterrorism, use data from a variety of sources (hospitals, clinics, laboratories) for aggregation, analysis and investigation [1-3]. For the detection of spatial clustering among disease cases, these aggregation systems achieve optimal detection sensitivity and specificity when using the most complete, accurate patient location data [4].

We have previously described a spatial de-identification algorithm that blurs precise point locations for patients, moving them a randomized distance according to a 2-dimensional Gaussian distribution with variance inversely proportional to the square of the underlying population density [5]. Other spatial anonymization approaches that have been employed include random skews, affine transformations, data aggregation techniques, and the use of software agents to preserve confidentiality [6,7]. Anonymization of patient address data by reassignment of geographic coordinates allows privacy preservation while sharing data for disease surveillance or biomedical research [5]. As the volume of personally-identifiable health data that is electronically transmitted and published has consistently increased [8], so has the magnitude of the threat to privacy. Geographical information is particularly identifying; we have demonstrated that it is possible to correctly identify most home addresses even from low resolution point-maps commonly published in journal articles [9].

**Figure 1:**
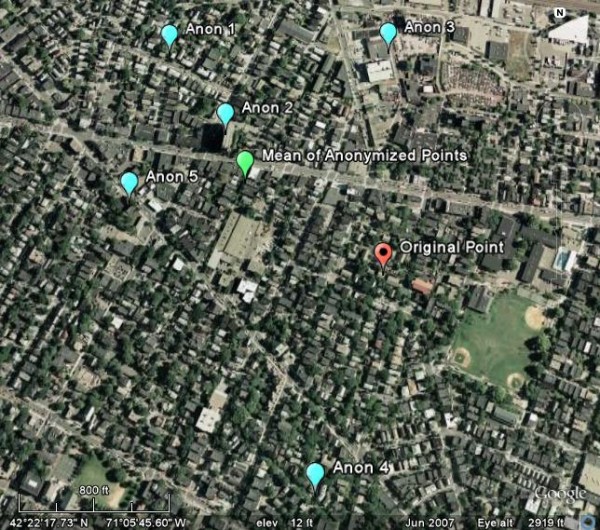
**Example of anonymized points that have been averaged**. An original data point (red) was anonymized using a population-density adjusted Gaussian skew algorithm five times (light blue points). Those points were averaged and the average coordinate value is plotted (green). The average of the anonymized points is nearer to the original point than each of the anonymized points.

We specifically explore whether de-identification algorithms that use spatial blurring – a non-deterministic process – may be susceptible to weakening when an adversary can access multiple anonymized versions of the same original data set [10]. For example, if data anonymized by a Gaussian blurring function were available upon request from a data source, the adversary could request anonymized patient data repeatedly. Since the data are non-deterministically anonymized, the results vary each time they are requested. By averaging the geocoded values for each visit, the anonymity afforded by the blurring algorithm may be reduced (Figure 1 illustrates the effect of averaging locations across the repeated anonymization passes to increase resolution for re-identification).

Here, we quantitatively demonstrate this vulnerability in two common anonymization approaches. We produce multiple anonymized data sets using a single set of addresses and then progressively average the anonymized results related to each address, characterizing the steep decline in distance of the re-identified point to the original location, (and the reduction in privacy) at each stage. Next, we propose and discuss two solutions to this specific class of vulnerabilities. The first tightly couples anonymization to a distributed health infrastructure that exchanges the data, so that it can control the number of copies distributed to any one party. The second is an extension to the spatial anonymization process employing a Markov process for increasing the anonymity of a 2-dimensional data set anonymized by Gaussian skew.

## Results

### Re-Identification of points anonymized using Gaussian and randomized skew

Additional information was ascertained from multiple anonymized copies of one original set of point locations, significantly weakening the anonymization used. The average distance to the original addresses after one anonymization pass, which represents the previously described [5] use of an anonymizing algorithm, was 0.69 km. After each point was inferred using the average of fifty Gaussian skew anonymization passes, the mean distance from the average of all of the anonymized points to the original point in the data set was reduced to 0.1 km.

Similarly, when the anonymizing algorithm is a uniform skew (a random skew that involves moving a point randomly within a square), re-identification attempts using the average of several anonymized data sets also reduced data set privacy markedly. The average distance to the original addresses after one anonymization pass, the traditional use of such algorithms, was set at 0.69 km, to match the level of skew used in the 2-dimensional Gaussian data sets. As in the case of the 2-dimensional Gaussian skew, the average distance to the original point was also reduced to just under 0.1 km after averaging 50 anonymized data sets.

**Figure 2:**
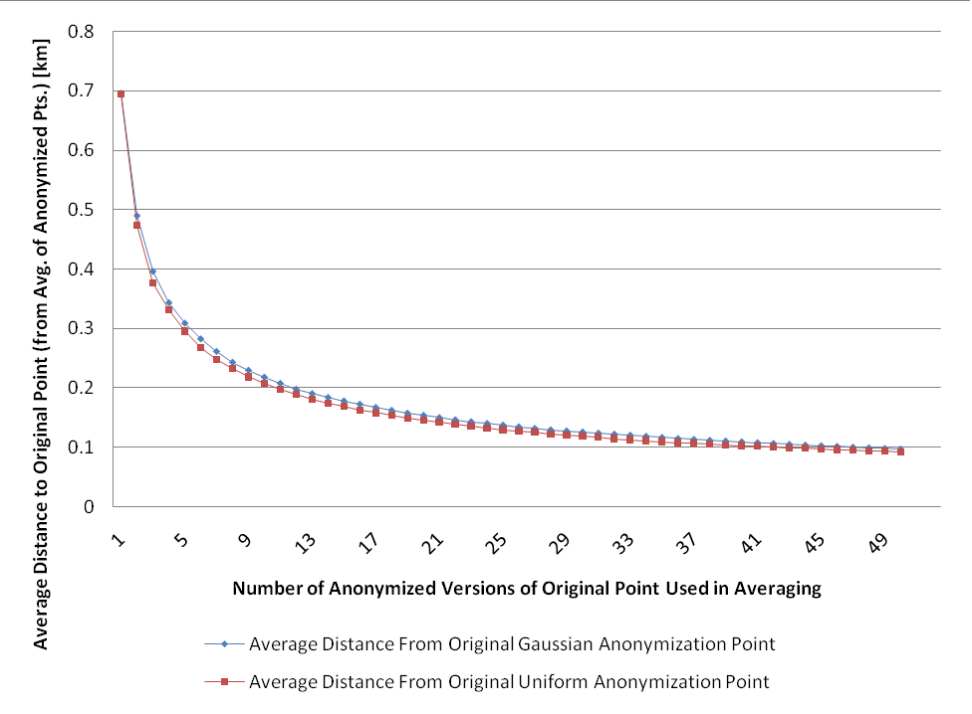
**Average distance to original point vs. number of anonymization versions**. The average distance to original point [km] vs. number of anonymization versions used in averaging is plotted for both Gaussian and uniform skew.

The average distance to the original address is plotted as a function of the number of separate anonymization passes used in the re-identification inference, for both anonymization methods in Figure 2. Attempts at inferring the original addresses using multiple anonymization passes, show that the average distance inversely varies with the square root of the number of anonymized data sets used in the inference. There is a sharp decrease in the average distance to the original address with 10 anonymization passes and thus a sharp decrease in data set anonymity.

## Discussion

### Re-identification of data anonymized with Gaussian and randomized skew

Re-anonymizing a single patient located at $\left(\begin{array}{c} x_{o}\\ y_{o} \end{array}\right)$*n *different times using Gaussian skew is equivalent to observing a sequence *L*_1_, *L*_2_, ..., *L*_*n *_of independent, identically distributed two-dimensional Gaussian random variables (all having the same probability density function). The average of these *n *observations

(2) $$\frac{\sum_{i=1}^{n}L_{i}}{n},$$

is itself a two-dimensional Gaussian random variable with mean $\left(\begin{array}{c} x_{o}\\ y_{o} \end{array}\right)$ and covariance matrix

$$\left[\begin{array}{cc} \frac{\sigma^{2}}{n} & 0\\ 0 & \frac{\sigma^{2}}{n} \end{array}\right].$$

In other words, the x- and y-coordinates are independent Gaussian random variables, each having a standard deviation of *σ*/$\sqrt{n}$. Hence, by taking the average of the anonymization passes, one can obtain the equivalent of a single anonymization pass under a less stringent Gaussian skew anonymization strategy with standard deviation *σ*/$\sqrt{n}$; for 100 passes, reducing the skewing standard deviation along each axis by a factor of 10.

In the uniform skew anonymization procedure, a patient at $\left(\begin{array}{c} x_{o}\\ y_{o} \end{array}\right)$ is moved with equal probability to any position in the square [*x*_0 _- *λ*, *x*_0 _+ *λ*]·[*y*_0 _- *λ*, *y*_0 _+ *λ*]. The new position is thus a two-dimensional uniform random variable, with mean $\left(\begin{array}{c} x_{o}\\ y_{o} \end{array}\right)$ and covariance

$$\left[\begin{array}{cc} \frac{\lambda^{2}}{3} & 0\\ 0 & \frac{\lambda^{2}}{3} \end{array}\right].$$

By the central limit theorem,

$$\frac{\sum_{i=1}^{n}L_{i}}{n}$$

is approximately normally distributed with mean $\left(\begin{array}{c} x_{o}\\ y_{o} \end{array}\right)$, and covariance matrix

$$\left[\begin{array}{cc} \frac{\lambda^{2}}{3n} & 0\\ 0 & \frac{\lambda^{2}}{3n} \end{array}\right].$$

Hence as the number of observations increases, the average of the observations tends to fall nearer to the original point.

It is important to note that while the difference in change to the covariance matrices would appear to make the Gaussian and uniform anonymization skews similar in their ability to protect privacy, this is not necessarily the case. The quantifiable estimate of anonymity, *k*-anonymity (a metric for data set privacy where *k *is the number of people among whom a specific individual cannot be distinguished [11]), that is achieved by each method is different – the Gaussian skew yields higher levels of *k*-anonymity than the randomized skew does with respect to the average distance moved for each case in a data set. We previously described a method for estimating spatial *k*-anonymity [5].

One might fear that an adversary could do even better, devising a novel strategy that uses the sequence *L*_1_, *L*_2_, ..., *L*_*n *_to get even closer to the original point $\left(\begin{array}{c} x_{o}\\ y_{o} \end{array}\right)$ than a Gaussian with this reduced variance; however, this is not possible without additional data. Stein showed that given *n *observations of a two-dimensional Gaussian random variable, the most efficient estimator of the mean of the Gaussian is simply the average of the points [12]. Although this seems intuitive for two dimensions, it is surprisingly not the case for three and higher dimensions [12,13].

### Anonymizing within a distributed network or health information exchange

**Figure 3:**
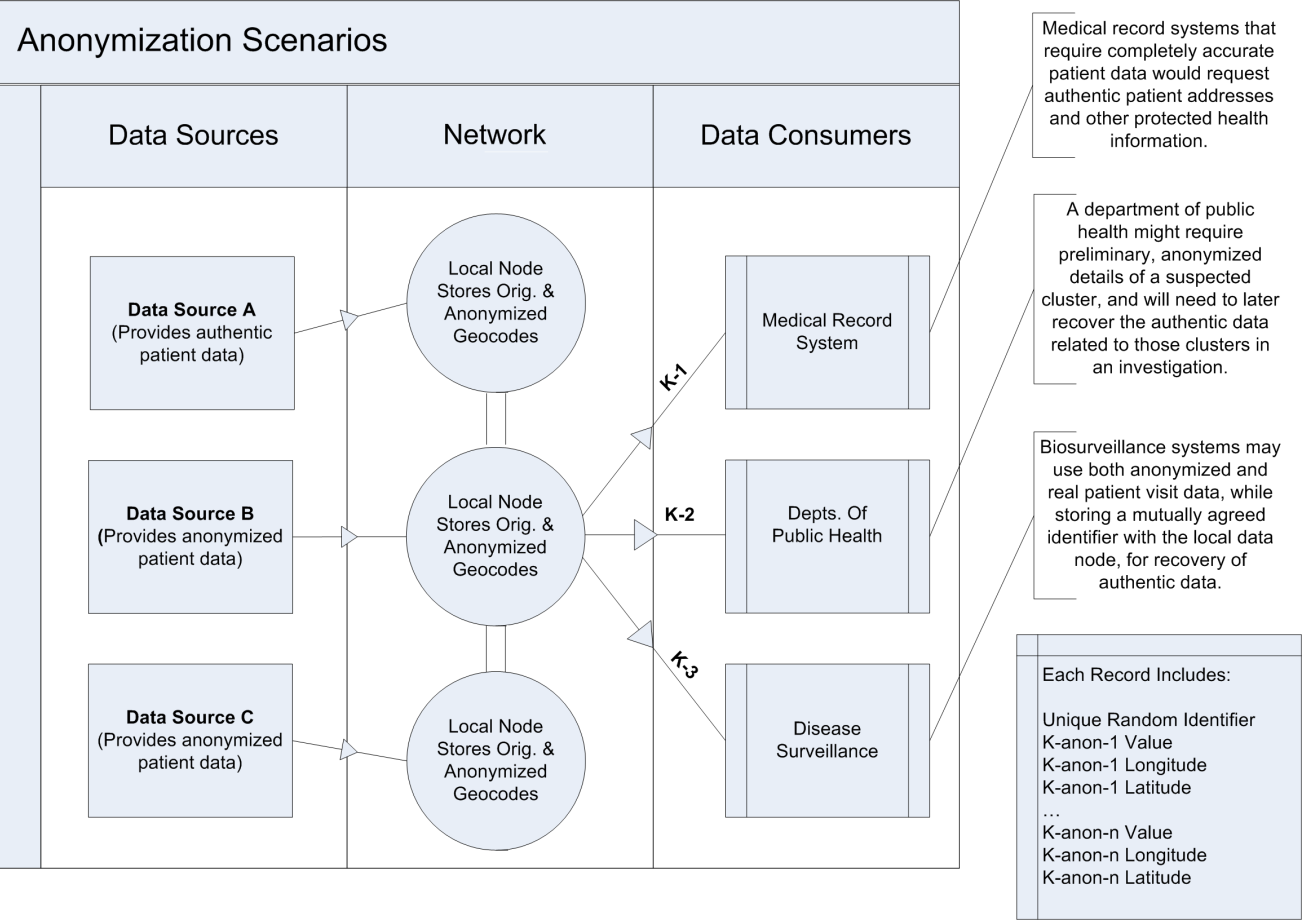
**Integration of anonymization within distributed EMR infrastructure**. Integration with a distributed electronic medical record infrastructure: a distributed data provisioning system provides anonymized spatial address data to three data consumers at three distinct *k*-anonymity privacy levels.

We believe that these results make a compelling case for infrastructure to control disclosure of anonymized data, so that the risk of this vulnerability is reduced. In Figure 3, we show an infrastructural solution for integrating anonymization into a distributed network that transmits health data. Ideally, data sources – and even patients – would be able to set a preferred level of data disclosure for a number of different purposes including research studies that integrate their clinical data, outcomes and public health surveillance. A data provisioning system could then distribute data to consumers at a variety of anonymized levels, under a clear set of policies and authorization requirements.

### Removing other identifying information from data sets to avoid re-linking

The vulnerability described in this paper relies on the ability to link anonymized data sets together using additional identifiers, or other demographic or clinical data. One possible solution is to swap the addresses in a given data set so that they are effectively unlinked with any unique clinical fields or identifiers, such as a medical record number. This unlinking of spatial data from unique identifiers, however, poses additional challenges: unlinking from any demographic identifiers could reduce the ability to conduct informative disease surveillance, or worse, could make it difficult to actually uncover the addresses of clustered cases when necessary, which is certainly a priority for a public health investigation.

This can be mitigated through the use of randomly generated identifiers for each anonymized instance of a specific record, stored for use in re-linking anonymized data with original data. Additionally, when attempting to determine correlates or predictors of disease, these additional fields may prove important for group stratification. With knowledge of the specific anonymization algorithm and background knowledge such as regional demographic data, it may be possible to further weaken some anonymization algorithms even without repeated attempts.

When considering only two dimensional geographical data, the best way to estimate original locations from several anonymized versions of the same original data set is to average the anonymized longitudes and latitudes. However, there are even more advanced re-identification techniques that can be used to improve the resolution of cases in practice, using data sets with three or more dimensions. If additional fields or identifiers are included in the data set, and those fields are in any way not randomly distributed (anything other than a randomly generated identifier), their presence has the potential to help achieve a higher resolution on the spatial coordinates, even if they do not contain geographical information. This is because there may be additional implicit information linked with spatial addresses in the other dimensions (or fields) that can lend intuition about the distribution of the anonymized spatial coordinates, using approaches that are more advanced than averaging of the fields to estimate the original location [13].

### Increasing anonymity using an algorithm based on a Markov process

**Figure 4:**
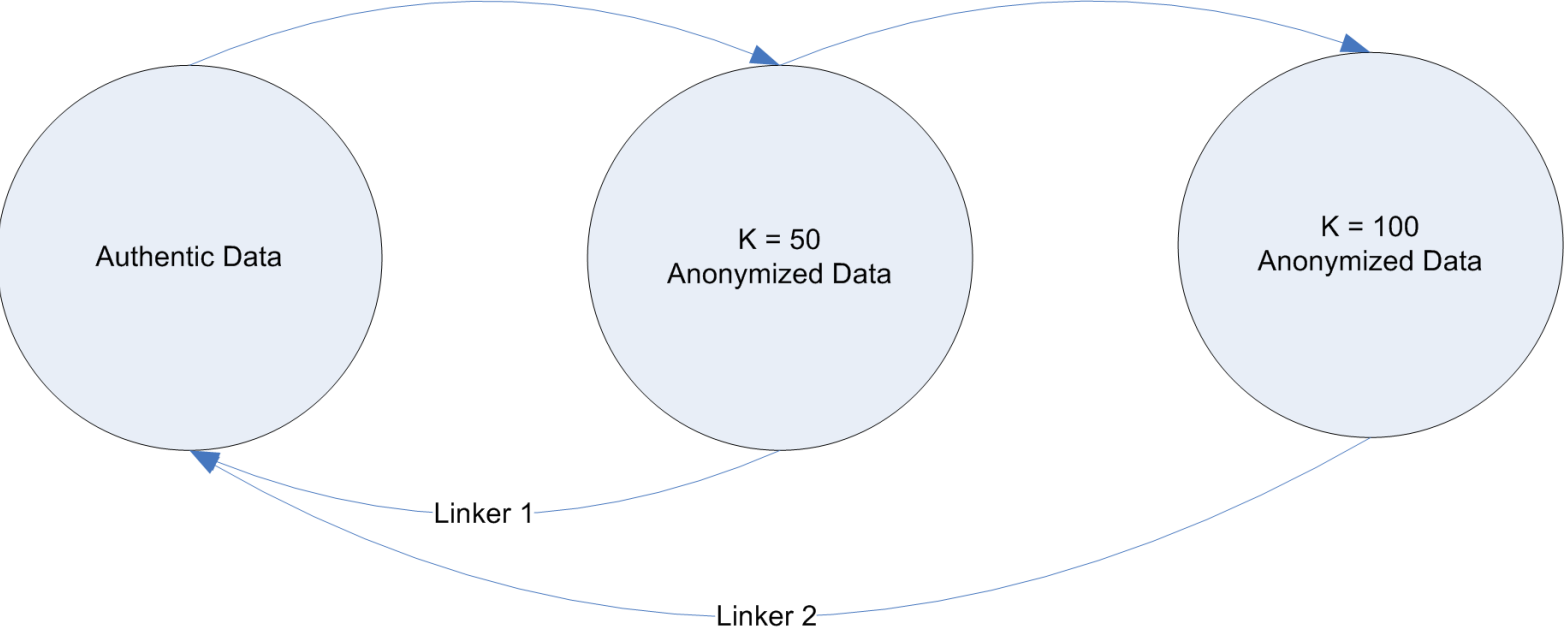
**Markov anonymization process to increase data set anonymity**. Markov processes to increase the anonymity level in a data set: an increase in the anonymity level of a data set, for example, increasing from k = 50 to k = 100, could be achieved by increasing the skew level of the k = 50 data set without knowledge of the authentic data. If increases are done in this way, the risk of a reverse identification attempt using averaging can be avoided.

As shown in Figure 3, one possible anonymization scenario is the sharing of data at a variety of privacy levels with different data consumers. To prevent privacy degradation by averaging when sharing data at multiple levels of *k*-anonymity [11], a Markov state process can be used to successively generate increasingly anonymized versions of the data set. The Markov property guarantees that several versions anonymized this way cannot be used to infer additional information about a patient's location. One example might be the need to provide multiple versions at two anonymized levels, one at *k *= 50 and another at *k *= 100. If the anonymization process is restricted to increasing the anonymization level to *k *= 100 by increasing the skew level from the *k *= 50 data set, and *not *from the original data set, there will be no way to decrease the privacy below the *k *= 50 level, simply by averaging the two data sets. This is illustrated in a Markov process model in Figure 4.

While infrastructure for controlled exchange of anonymized health data protects against some vulnerabilities, there are still other methods that could reduce the privacy level of a data set. For example, it is still possible to gain insight into the actual distribution of cases anonymized with knowledge of physical boundaries, highly constrained patient distributions, or other clinical or demographic information about cases. Further study is needed to adequately constrain the anonymized geographical distributions of cases such that this risk is minimized.

## Conclusion

In order to protect privacy when using spatial skew algorithms, the number of distinct anonymization results or passes that represent the same data must be controlled. Limiting the generation or disclosure of more than one version will avoid re-identification through averaging. Alternative approaches include integration of anonymization into data provisioning systems to achieve such a restricted data release, or the use of a Markov process to generate multiple anonymized data sets of the same records. These approaches avoid running the algorithm anew with each request, reducing the variation that is at the root of the vulnerability.

## Methods

### Geographical test data sets

**Figure 5:**
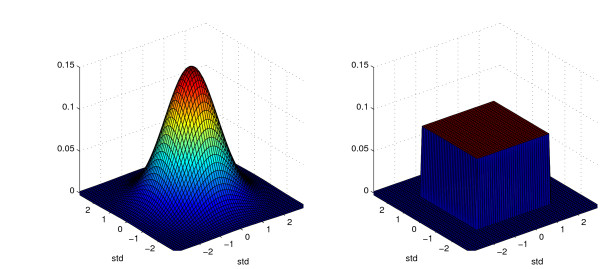
**Anonymization algorithm translation probability density functions**. Probability distribution functions for the two anonymization methods, 2-dimensional Gaussian skew (left) and uniform skew (right).

A data set containing artificially-generated geocoded values for 10,000 sample patients was created using a spatial cluster creation tool [14,15]. All points were uniformly distributed within a circle of radius 800 m centered in Boston, MA, and assigned a unique numeric identifier for tracking. Each of the geocoded addresses was then anonymized using a Gaussian 2-dimensional spatial blur skew that was adjusted for population density [5], fifty separate times. A second anonymization approach, a uniform skew, was used to create a second group of 50 anonymized data sets. Each geocode that was anonymized using the uniform skew method was moved a distance, in meters, ranging from [-*λ*, *λ*], independently in each dimension. Figure 5 describes the 2-dimensional probability distribution function for both of these anonymization algorithms.

### Population-adjusted 2-dimensional Gaussian skew

In the simplest case, the Gaussian skew anonymization procedure is a probabilistic strategy that reassigns an original point, with coordinates $\left(\begin{array}{c} x_{o}\\ y_{o} \end{array}\right)$, to a new location based on two Gaussian probability density functions

(1) $$\begin{array}{cc} f_{X}(x)=\frac{1}{\sqrt{2\pi }\sigma_{x}}e^{\frac{-{\left(x-x_{0}\right)}^{2}}{2\sigma_{x}^{2}}}, & f_{Y}(y)=\frac{1}{\sqrt{2\pi }\sigma_{y}}e^{\frac{-{\left(y-y_{0}\right)}^{2}}{2\sigma_{y}^{2}}}. \end{array}$$

These are simply 1-dimensional Gaussians with means equal to the original coordinates *x*_0 _and *y*_0_, respectively, and standard deviations *σ*_*x *_and *σ*_*y*_. The parameters *σ*_*x *_and *σ*_*y *_are proportional to the desired level of anonymity *k*, and are inversely proportional to the population density at $\left(\begin{array}{c} x_{o}\\ y_{o} \end{array}\right)$. In other words, the greater the anonymity desired, or the lower the underlying population density, the farther points are moved on average.

### Re-identification through averaging

**Figure 6:**
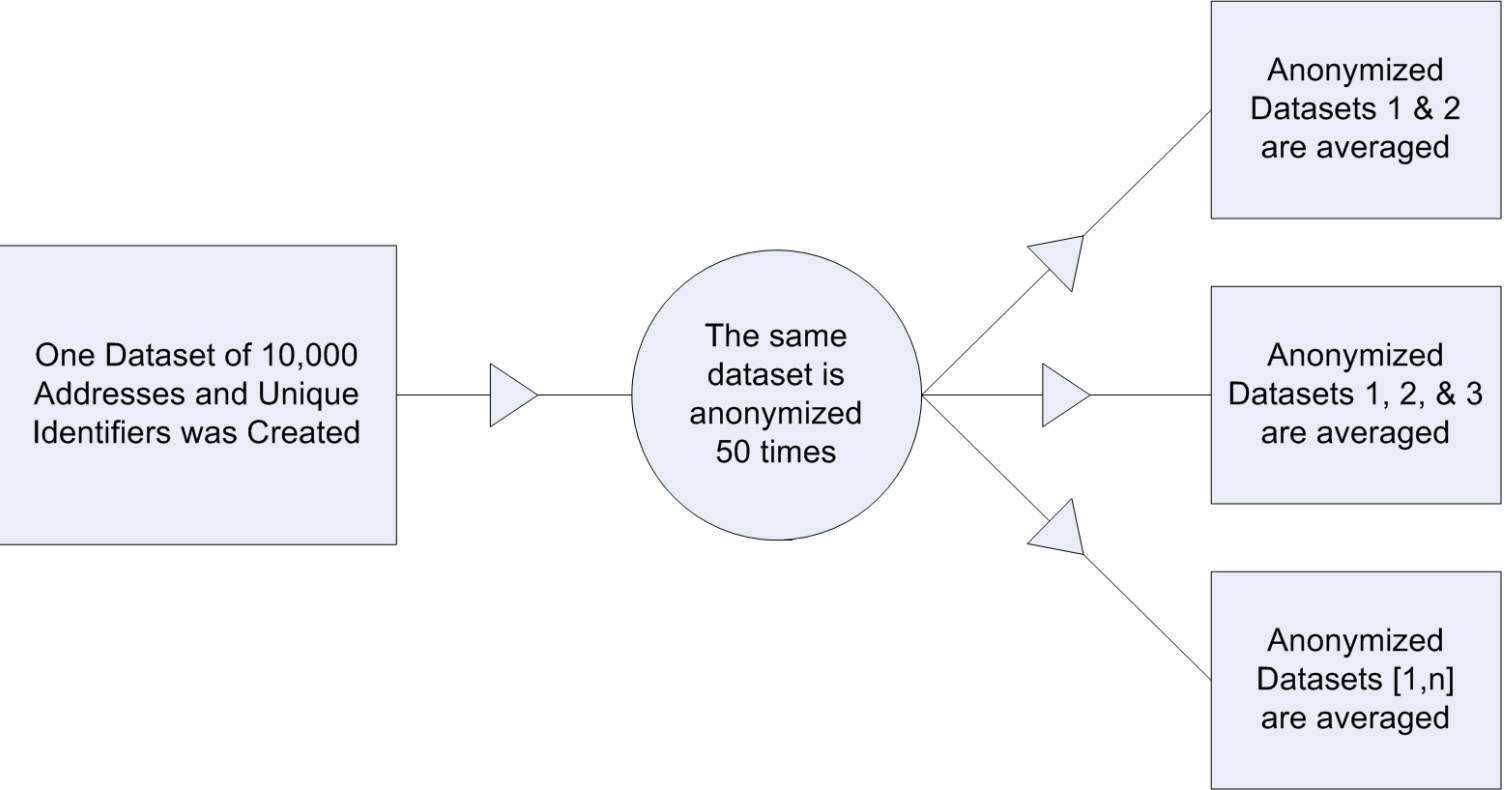
**Experimental methods design**. One data set of 10,000 artificially generated case locations and unique identifiers were created. The data set was anonymized 50 times using a 2-dimensional Gaussian-based skew, and 50 times using a 2-dimensional uniform skew.

With each subsequent anonymized version, the geocoded points that referred to the same individual address were averaged to estimate the original address. For re-identification inference number *n*, the anonymized versions of the same address were averaged from data sets [1, *n*], as shown in Figure 6. For example, the second re-identification inference data set included the averages of addresses from anonymized data sets 1 and 2, the third inference data set included anonymized data from data sets 1, 2, and 3, and so on. After each pass, the distance between the average anonymized point and the original address was calculated.

## Competing interests

The authors declare that they have no competing interests.

## Authors' contributions

CAC participated in the study concept and design, acquisition of data, analysis and interpretation of data, statistical analysis, drafting and revision of the manuscript. SCW participated in the analysis and interpretation of data, statistical analysis, drafting and revision of the manuscript. KDM participated in the analysis and interpretation of data, study supervision, and critical revision of manuscript. All authors read and approved the final manuscript.
